# MicroRNA-5195-3p alleviates high glucose‑induced injury in human ARPE-19 cells by targeting GMFB

**DOI:** 10.1371/journal.pone.0260071

**Published:** 2021-11-18

**Authors:** Jingjing Liu, Yongsheng Hou, Lili Lin, Nannan Yu, Yanyan Zhang

**Affiliations:** Eye Hospital, the First Affiliated Hospital of Harbin Medical University, Harbin, Heilongjiang, China; University of Florida, UNITED STATES

## Abstract

Hyperglycemia is generally considered to be an important cause of diabetic retinopathy (DR). The aim of the present study was to investigate the role of miR-5195-3p in high glucose (HG)-induced human retinal pigment epithelial ARPE-19 cell injury. Here, we first found that the expression level of miR-5195-3p was significantly downregulated in HG-stimulated ARPE-19 cells using reverse transcription quantitative PCR. Overexpression of miR-5195-3p attenuated the impaired cell viability, increased apoptosis and pro-inflammatory cytokines secretion in ARPE-19 cells under HG condition using CCK-8 assay, flow cytometry and ELISA assay, respectively. Luciferase reporter assay showed that miR-5195-3p could specifically bind to the 3’UTR of glia maturation factor-β (GMFB). GMFB overexpression reversed, while knockdown enhanced the protective effects of miR-5195-3p overexpression against HG-induced ARPE-19 cell injury. In summary, miR-5195-3p targeting GMFB might be a potential therapeutic target for DR.

## Introduction

Diabetic retinopathy (DR) as a major complication of diabetes is one of the leading causes of vision loss and even blindness, which is characterized by retinal edema, neuronal dysfunction and breakdown of the blood–retinal barrier (BRB) [1,2]. Hyperglycemia as the main cause of the DR development can cause pathological metabolism and biochemical changes [3,4]. Retinal pigment epithelial (RPE) cells, an important cellular component of the outer BRB, could selectively control the flux of molecules into and out of the retina, which are most vulnerable to hyperglycemia [5–7]. Accumulating evidence has indicated the RPE dysfunction is particularly relevant to the development of DR [8,9]. Nevertheless, the molecular mechanisms underlying hyperglycemia-associated injures in RPE have not been fully elucidated.

MicroRNAs (miRNAs/miRs) are a group of single-stranded, short (∼22 nucleotide) and noncoding RNA molecules that are capable of negatively regulating gene expression by binding to the seed region in the 3′-untranslated regions (3′-UTRs) of target mRNAs [10,11]. Studies have found that miRNAs are implicated in a broad range of biological processes, such as cell growth, differentiation and apoptosis [12], which have been gradually revealed to participate in the pathogenesis of various diseases, including diabetes and diabetic complications [13,14]. Moreover, a very recent study has shown that a series of miRNAs, including miR-20a-5p, miR-20b and miR-27a-5p were dysregulated in the retina and serum of diabetic mice and patients as well [15], which demonstrated the important link between miRNA expression and DR. Transforming growth factor β1 (TGFβ1) is a proinflammatory cytokine that has been implicated in the pathogenesis of DR, particularly in the late phase of disease [16]. Recently, miR-5195-3p, a newly discovered member of the miRs family, has been reported to exert its suppressive activity in HCT116 cells by modulating TGFβ/SMAD signaling [17]. In addition to the role of miR-5195-3p in proinflammation, miR-5195-3p has been widely reported to be associated with cell growth, proliferation and apoptosis. For example, miR-5195-3p suppressed growth and proliferation of human bladder cancer cells via suppression of Krüppel-like factor 5 (KLF5) [18]. MiR-5195-3p upregulation repressed cell proliferation by targeting MYO6 in non-small cell lung cancer [19]. Similarly, the regulatory role of miR-5195-3p on decreased cell proliferation and increased apoptosis was also demonstrated on osteosarcoma [20], glioma [21] and hepatocellular carcinoma [22]. Therefore, we speculated that miR-5195-3p might play an important role in DR progression via regulating hyperglycemia-induced cell inflammation and apoptosis.

Glia maturation factor, as a growth and differentiation factor, is actually a mixture of two compounds (glia maturation factor-β (GMFB) and GMF-γ) [23]. GMFB is located on the long arm of human chromosome 14 with a molecular weight of 7 kb in length, which is associated with apoptosis, oxidative stress and neuroinflammation [24,25]. The expression of GMFB has been shown to be upregulated during some pathological conditions, including ovarian cancer [26], Alzheimer’s disease [27] and Parkinson’s disease [28]. Interestingly, a recent study by Xu et al [29] showed that GMFB might be an initiator of the lung injury induced by acute cerebral ischemia by presenting evidences that rat primary astrocytes cultures containing recombinant GMFB showed increased levels of reactive oxygen species and a deterioration in the state of the cells. GMFB has been found to be upregulated in several neuroinflammation and neurodegeneration conditions [25]. Moreover, we observed that GMFB was a target gene of miR-5195-3p by Targetscan online prediction. Based on these evidences, we supposed that miR-5195-3p/GMFB axis might be associated with the biological behaviors of RPE cells involved in DR pathogenesis.

In this study, we treated human RPE cell line ARPE-19 with high glucose (HG) to construct a cell model of DR in vitro. Then, we explored the effects of miR-5195-3p and GMFB on HG-induced cell viability, inflammation and apoptosis. Moreover, we explored the possible relationship between miR-5195-3p and GMFB and further investigated whether GMFB was involved in miR-5195-3p regulating HG-induced ARPE-19 cell injury.

## Materials and methods

### Cell culture and treatment

Human retinal pigment epithelial (RPE) cell line ARPE-19 (Cat# 2302) was purchased from American Type Culture Collection (ATCC; Manassas, VA, USA) and cultured in Dulbecco’s Modified Eagle’s Medium (DMEM)/F-12 medium supplemented with 10% (v/v) fetal bovine serum (FBS, Gibco, Thermo Fisher Scientific Inc., Waltham, MA, USA) in a humidified atmosphere containing 5% CO_2_ at 37°C. Once the cells reached approximately 85% confluence, cells were incubated with normal glucose (NG; 5.5 mmol/L) as a control or with high glucose (HG; 25 mmol/L) for 24 h to simulate the in vitro DR cell model. Every 2–3 days, the culture medium was changed to eliminate metabolic byproducts.

### Reverse transcription quantitative PCR

Total RNA sample was isolated from cells with TRIzol reagent (Invitrogen; Thermo Fisher Scientific, Inc.) and reversed to cDNA using a PrimeScript reverse transcription reagent kit (Takara Biotechnology Co., Ltd.) according to the manufacturer’s protocols. The expression of miR-5195-3p was determined using the a miScript SYBR Green PCR Kit (Thermo Fisher Scientific, Inc.) on an ABI 7500 thermal cycler (Applied Biosystems, USA) with the thermocycling conditions (Initial denaturation at 95°C for 5 min and 40 cycles of denaturation at 95°C for 10 sec, annealing at 60°C for 10 sec, and extension at 72°C for 30 sec.). The specific primers for miR-5195-3p and U6 were listed as follows: miR-5195-3p, forward: 5′-TAGCAGACTCTTATGATG-3′ and reverse: 5′-TGGTGGAGTCGTCGTG-3′; U6, forward: 5′-CTCGCTTCGGCAGCACA-3′ and reverse: 5′-AACGCTTCACGAATTTGCGT-3′. Relative expression of miR-5195-3p was calculated by the 2^–ΔΔCT^ method and normalized to the expression of U6.

### Western blot analysis

Extraction of total protein sample from cultured cells was performed using radioimmunoprecipitation assay buffer (Beyotime Institute of Biotechnology, Shanghai, China) and protein concentration was measured using a BCA Protein Assay kit (Beyotime Institute of Biotechnology) according to the manufacturer’s protocol. Equal amount protein sample (30 μg) was subjected to 10% SDS-PAGE electrophoresis and transferred onto polyvinylidene difluoride (PVDF) membranes (Millipore, Billerica, MA, USA). Then, the membrane was blocked with 5% skimmed milk at room temperature for 2 h. After washed with phosphate buffered saline Twen-20 (PBST) three times, the membranes were incubated with primary antibodies against GMFB and glyceraldehyde-3-phosphate dehydrogenase (GAPDH, Abcam, Cambridge, UK) at 4°C overnight, followed by incubation with horseradish peroxidase-conjugated secondary antibody (Cell Signaling Technology, Inc., Danvers, MA, USA) at room temperature for 2 h. Subsequently, the target proteins were visualized with an Enhanced Chemiluminescence (ECL) Plus Kit (Millipore, USA) and quantitatively analyzed with ImageJ software.

### Cell transfection

The miR-5195-3p mimics, miRNA negative control (miR-NC), small interfering RNA targeting GMFB (si-GMFB) and small interfering negative control (si-NC) were designed and synthesized by GenePharma Co. Ltd (Shanghai, China). The open reading frame of GMFB without 3’UTR was inserted into the pcDNA3.1 vector (Sangon Biotech, Shanghai, China) to generate pcDNA3.1-GMFB vector. The empty pcDNA3.1 vector was used as control group. ARPE-19 cells were plated in six-well plates (1 × 10^6^ cells per well) on the day prior to transfection. Then, ARPE-19 cells were transfected with miR-5195-3p mimics, miR-NC, si-GMFB or si-NC, followed by 24 h incubation with HG. In the rescue experiments. ARPE-19 cells were co-transfected with miR-5195-3p mimics and pcDNA3.1-GMFB or si-GMFB, followed by 24 h incubation with HG. All transfection protocols were performed with Lipofectamine 2000 (Invitrogen) according to the instructions from the manufacturer.

### Dual-luciferase reporter assay

We performed bioinformatics analysis via the TargetScan version 7.1 online tool (http://www.targetscan.org/vert_71/) to predict the possible miR-5195-3p binding sites in the GMFB gene 3′-UTR. Next, the 3′-UTR of human GMFB containing the predicted wild type (WT) binding sites or mutant type (MUT) binding sites was amplified and cloned into a psiCHECK-reporter plasmid (Promega) and then validated through DNA sequencing. Subsequently, cells were co-transfected with the reporter plasmids (WT GMFB or MUT GMFB) and miR-5195-3p mimics or miR-NC using Lipofectamine 2000 (Invitrogen). Relative luciferase activities were measured at 48 h after transfection with the Dual-Luciferase reporter Assay System (Promega, Madison, WI, USA).

### Cell viability assay

The cell viability of ARPE-19 cells from different groups was determined by performing Cell Counting Kit-8 (CCK-8) assay (Dojindo, Tokyo, Japan). In brief, cells at a density of 4 × 10^3^ cells per well were seeded in a 96-well plate and cultured overnight at 37°C. At the incubation for intended periods (0, 24, 48 and 72 h), cells were incubated with 10 μL CCK-8 solution for another 2 h at 37°C, followed by measurement of optical density (OD) value using a microplate reader at 450 nm.

### ELISA assay

ARPE-19 cells from different groups were seeded into 96-well plates at a density of 1 × 10^5^ cells per well. After centrifuged at 6,000 × g for 15 min at 4°C, the supernatant from cell culture was collected and the release of IL-1β and TNF-α in supernatant was analyzed using commercially available ELISA assay kits according to the manufacturer’s instructions. The concentration of IL-1β and TNF-α was expressed as pictogram per milliliter (pg/ml) according to the standard curve.

### Cell apoptosis analysis

Apoptotic cells were analyzed with an Annexin V-APC/7-AAD Apoptosis Detection kit (KeyGen Biotech Co., Ltd., Nanjing, China) according to the manufacturer’s protocol. In brief, ARPE-19 cells from different groups were harvested and washed with ice-cold PBS for three times. After resuspended in 500 μl binding buffer, cells were incubated with 5 μL Annexin V-APC for 15 min in the dark and 5 μl 7-AAD for 5 min at room temperature. Finally, stained cells were analyzed for apoptosis (Annexin V-positive) by flow cytometry (FACSCalibur; BD Biosciences, Franklin Lakes, NJ, USA).

### Statistical analysis

All the in vitro experiments were performed in triplicate independently and data were expressed as mean ± standard deviation (SD). Statistical analysis was performed with GraphPad Prism version 6.0. Differences between two groups were compared with Student’s t test, while differences among groups were assessed by One-way or two-way ANOVA followed by Tukey’s post hoc test. Statistical significance was accepted when p-value less than 0.05.

## Results

### The expression and correlation of miR-5195-3p and GMFB in HG-induced ARPE-19 cells

**Fig 1:**
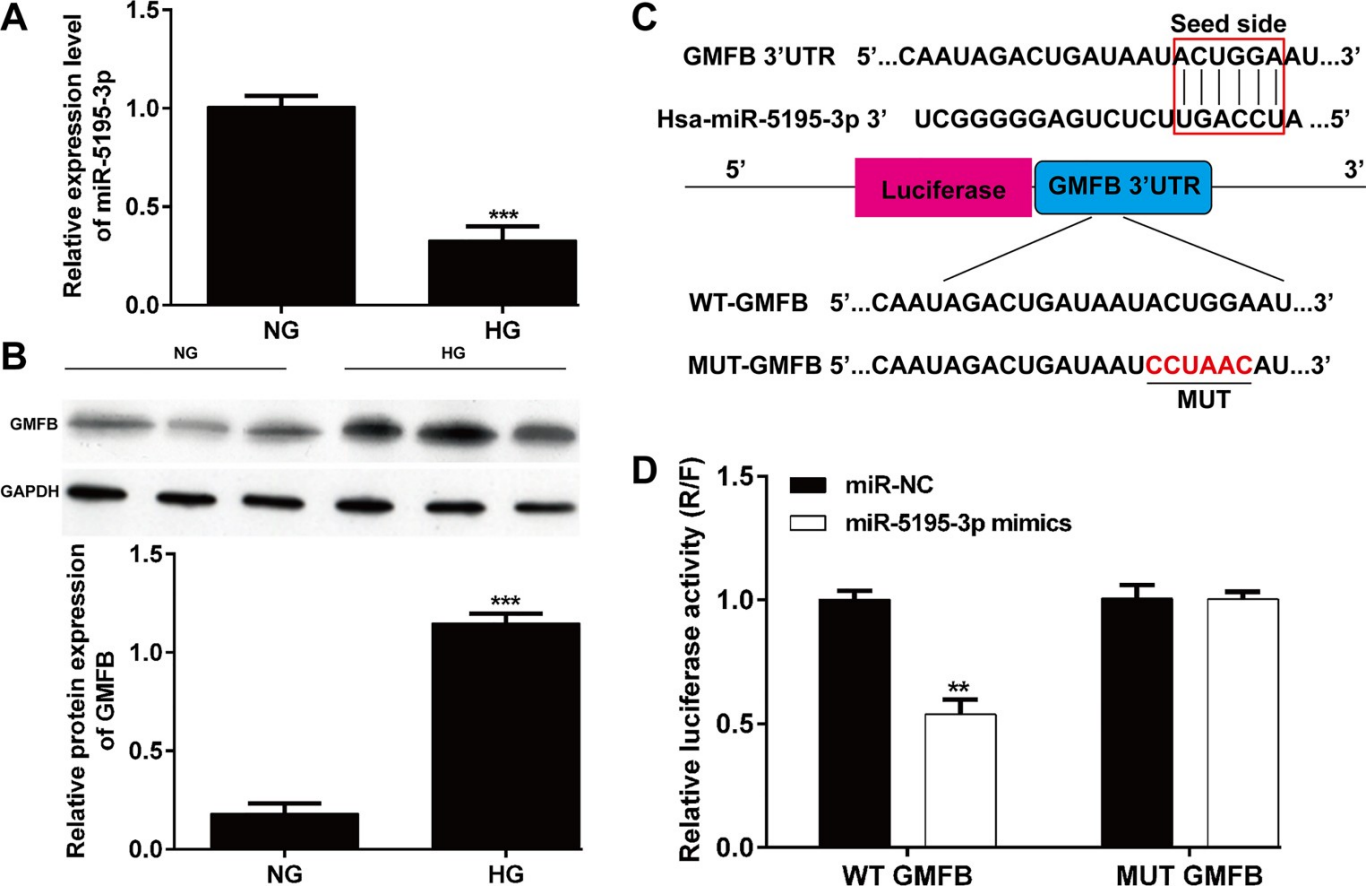
The expression levels and correlation of miR-5195-3p and GMFB. (A) In HG-induced ARPE-19 cells, miR-5195-3p was significantly downregulated compared with that of NG group. (B) GMFB protein expression was upregulated in HG-induced ARPE-19 cells compared with that of NG group. ***p < 0.001, compared with NG; (C) miR-5195-3p was predicted as a direct target of miR-5195-3p predicted by the miRNA online database (TargetScanHuman7.1). (D) Dual-luciferase reporter assay using ARPE-19 cells showed the direct interaction of miR-5195-3p and the 3′UTR of GMFB. After 48-h co-transfection, overexpression of miR-5195-3p reduced the luciferase activity of WT reporter but had no inhibition on the MUT reporter. Data were shown as mean ± SD. **p < 0.01 compared with miR-NC. NC, negative control; HG, high glucose; NG, normal glucose; WT, wild type; MUT, mutant type.

We first determined the expression of miR-5195-3p and GMFB in ARPE-19 cells under HG stimulation. The results from reverse transcription quantitative PCR showed that the expression of miR-5195-3p was significantly downregulated in ARPE-19 cells from HG group compared with that in NG group (**Fig 1A**). Western blot results indicated that GMFB protein expression was obviously increased after HG treatment in ARPE-19 cells compared with control group (**Fig 1B**). With the miRNA online prediction database (TargetScanHuman7.1), we found that GMFB was identified as a potential target of miR-5195-3p (**Fig 1C**). To further confirm this prediction, we constructed WT GMFB vector or MUT GBFM vector and applied a dual-luciferase reporter assay. As shown in **Fig 1D**, overexpressed miR-5195-3p reduced the luciferase activity of WT GMFB reporter but had no inhibition on the MUT GMFB reporter vector. The results of this study showed that GMFB might be the downstream target gene of miR-5195-3p.

### Overexpression of miR-5195-3p attenuated HG-induced ARPE-19 cell injury

**Fig 2:**
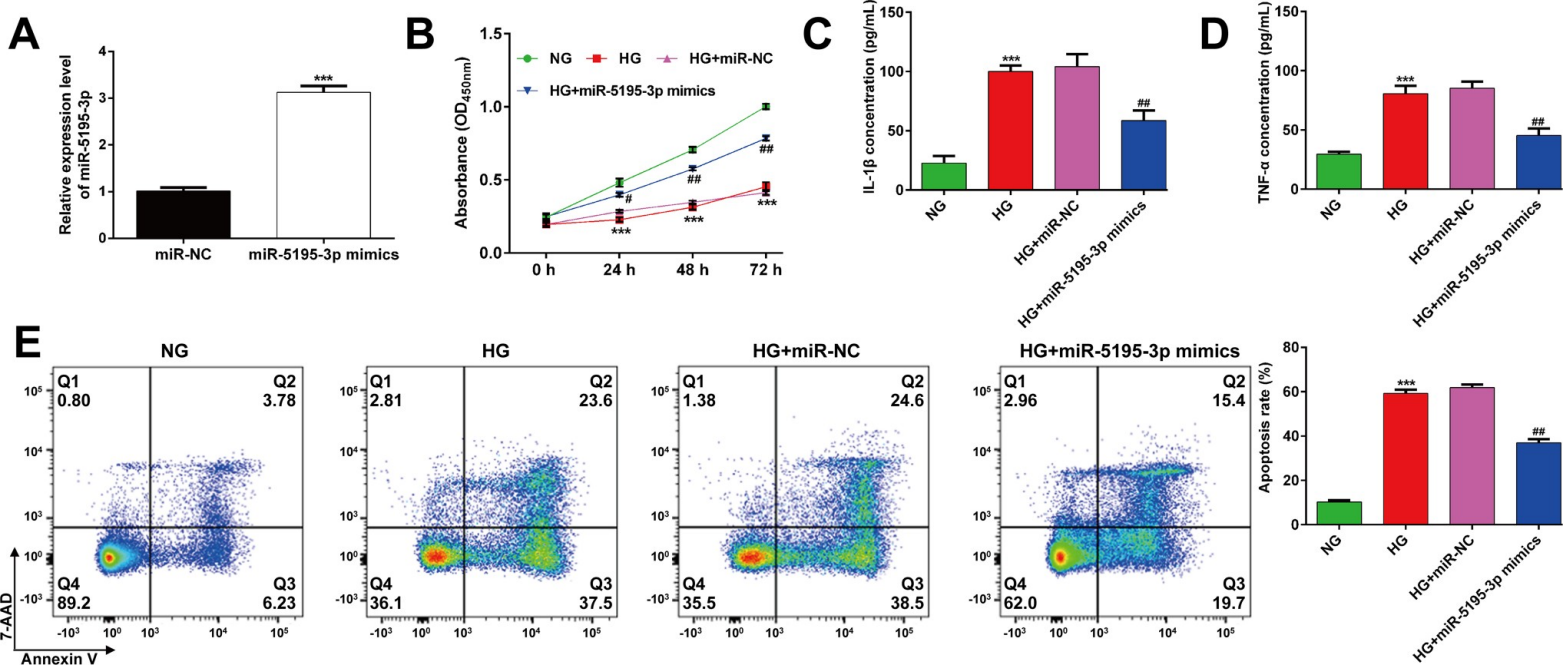
Effects of miR-5195-3p overexpression on ARPE-19 cell viability, inflammation and apoptosis after HG stimulation. (A) Analysis of reverse transcription quantitative PCR was conducted to evaluate miR-5195-3p expression in ARPE-19 cells transfected with miR-5195-3p mimics or miR-NC. ***p < 0.001, compared with miR-NC; (B) The transfected ARPE-19 cells were exposed to HG, followed by CCK-8 assay. ELISA assay was performed to analyze the release of IL-1β (C) and TNF-α (D) in transfected ARPE-19 cells, followed by HG stimulation. (E) The percentages of apoptotic cells were compared in transfected ARPE-19 cells, followed by HG stimulation. Data were shown as mean ± SD. ***p < 0.001 compared with NG; #*p* < 0.05, ##*p* < 0.01, compared with HG + miR-NC; NC, negative control; HG, high glucose; NG, normal glucose.

As the expression of miR-5195-3p was downregulated in HG-induced ARPE-19 cells, transfection with miR-5195-3p mimics was conducted in ARPE-19 cells to overexpress miR-5195-3p. As demonstrated by reverse transcription quantitative PCR, cells transfected with miR-5195-3p mimics had significantly elevated miR-5195-3p expression levels than cells transfected with miR-NC (**Fig 2A**). Transfected ARPE-19 cells were then exposed HG to mimic hyperglycemia insult, followed by measurements on cell viability, inflammation and apoptosis status. The results from CCK-8 assay showed that HG stimulation for 24 h significantly decreased ARPE-19 cell viability and this effect was partially attenuated by miR-5195-3p overexpression (**Fig 2B**). In the ELISA assay, it indicated that markedly increased releases of pro-inflammatory cytokines, including IL-1β and TNF-α was recovered in HG-stimulated ARPE-19 cells if they were transfected with miR-5195-3p mimics, as compared to cells transfected with miR-NC (**Fig 2C and 2D**). Moreover, flow cytometry analysis demonstrated that miR-5195-3p overexpression significantly suppressed the increased apoptotic ARPE-19 cells induced by HG stimulation (**Fig 2E**). These data suggested that miR-5195-3p overexpression restored cell viability and attenuated apoptosis and inflammation in HG-induced ARPE-19 cells.

### Knockdown of GMFB alleviated HG-induced ARPE-19 cell injury

**Fig 3:**
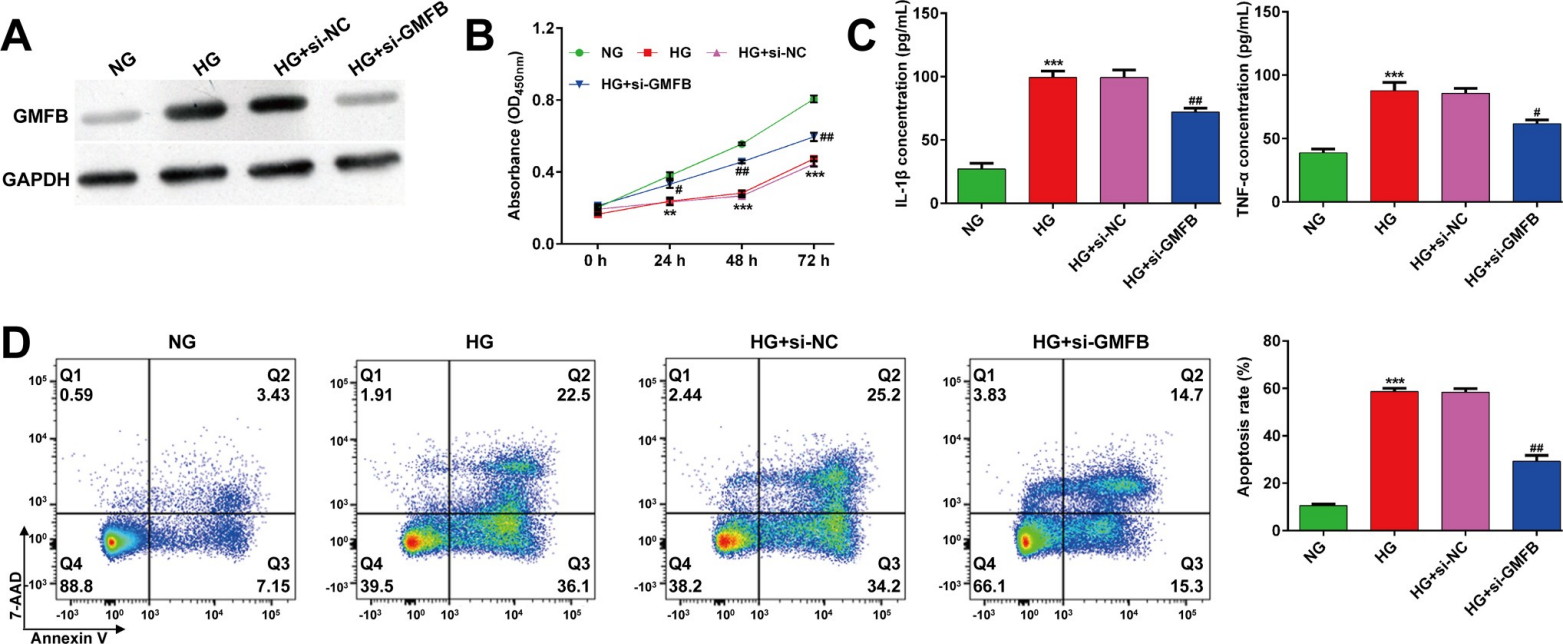
Effects of GMFB knockdown on ARPE-19 cell viability, inflammation and apoptosis after HG stimulation. (A) Western blot analysis was conducted to evaluate GMFB protein expression in ARPE-19 cells transfected with si-GMFB or si-NC. (B) The transfected ARPE-19 cells were exposed to HG, followed by CCK-8 assay. (C) ELISA assay was performed to analyze the release of IL-1β and TNF-α in transfected ARPE-19 cells, followed by HG stimulation. (D) The percentages of apoptotic cells were compared in transfected ARPE-19 cells, followed by HG stimulation. Data were shown as mean ± SD. **p < 0.01, ***p < 0.001, compared with NG; #*p* < 0.05, ##*p* < 0.01, compared with HG + si-NC; NC, negative control; HG, high glucose; NG, normal glucose; si, small interfering.

As the expression of GMFB was upregulated in HG-induced ARPE-19 cells, we performed loss-of-function assay in ARPE-19 cells by transfection with si-GMFB or si-NC. As shown in **Fig 3A**, si-GMFB transfection obviously suppressed the elevated GMFB protein expression in HG-induced ARPE-19 cells. CCK-8 assay showed that knockdown of GMFB significantly recovered the decreased ARPE-19 cell viability induced by HG stimulation (**Fig 3B**). Consistent with miR-5195-3p overexpression, HG-induced elevation of pro-inflammatory cytokines (IL-1β and TNF-α) (**Fig 3C**) and cell apoptosis (**Fig 3D**) in ARPE-19 cells was significantly alleviated after GMFB knockdown.

### Overexpression of GMFB reversed the protective effects of miR-5195-3p overexpression against HG-induced ARPE-19 cell injury

**Fig 4:**
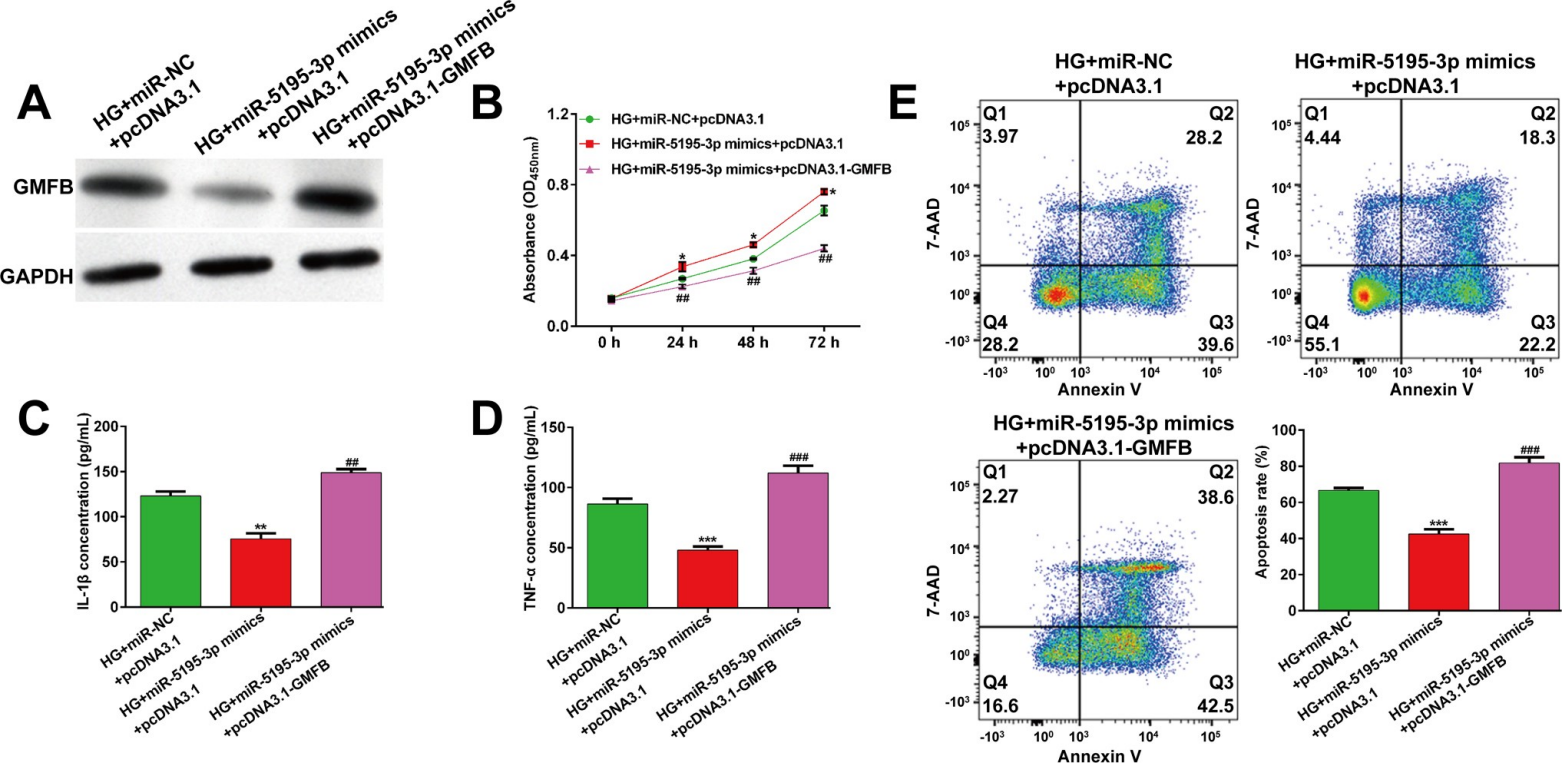
Overexpression of GMFB reversed the protective effects of miR-5195-3p overexpression against HG-induced ARPE-19 cell injury. ARPE-19 cells were co-transfected with miR-5195-3p mimics with either pcDNA3.1-GMFB or pcDNA3.1, followed by 24 h incubation with HG. (A) The protein expression of GMFB was detected by western blot analysis. (B) Cell viability was analyzed using CCK-8 assay. (C-D) ELISA assay was performed to analyze the release of IL-1β and TNF-α in transfected ARPE-19 cells, followed by HG stimulation. (E) The percentages of apoptotic cells were compared in transfected ARPE-19 cells, followed by HG stimulation. Data were shown as mean ± SD. *p < 0.05, **p < 0.01, ***p < 0.001, compared with HG + miR-NC + pcDNA3.1; ##*p* < 0.01, ###*p* < 0.001, compared with HG + miR-5195-3p mimics + pcDNA3.1; NC, negative control; HG, high glucose.

To investigate whether GMFB was involved in miR-5195-3p regulating HG-induced ARPE-19 cell injury, we performed rescue experiments in ARPE-19 cells by co-transfection with miR-5195-3p mimics and pcDNA3.1-GMFB, followed by 24h HG stimulation. As shown in **Fig 4A**, pcDNA3.1-GMFB transfection obviously recovered the inhibition of GMFB induced by miR-5195-3p mimics in HG-stimulated ARPE-19 cells. As expected, results from CCK-8 assay (**Fig 4B**), ELISA assay (**Fig 4C and 4D**) and flow cytometry analysis (**Fig 4E**) indicated that GMFB overexpression significantly reversed the effects of miR-5195-3p on cell viability, pro-inflammatory cytokines (IL-1β and TNF-α) and cell apoptosis in HG-stimulated ARPE-19 cells.

### Knockdown of GMFB enhanced the protective effects of miR-5195-3p overexpression against HG-induced ARPE-19 cell injury

**Fig 5:**
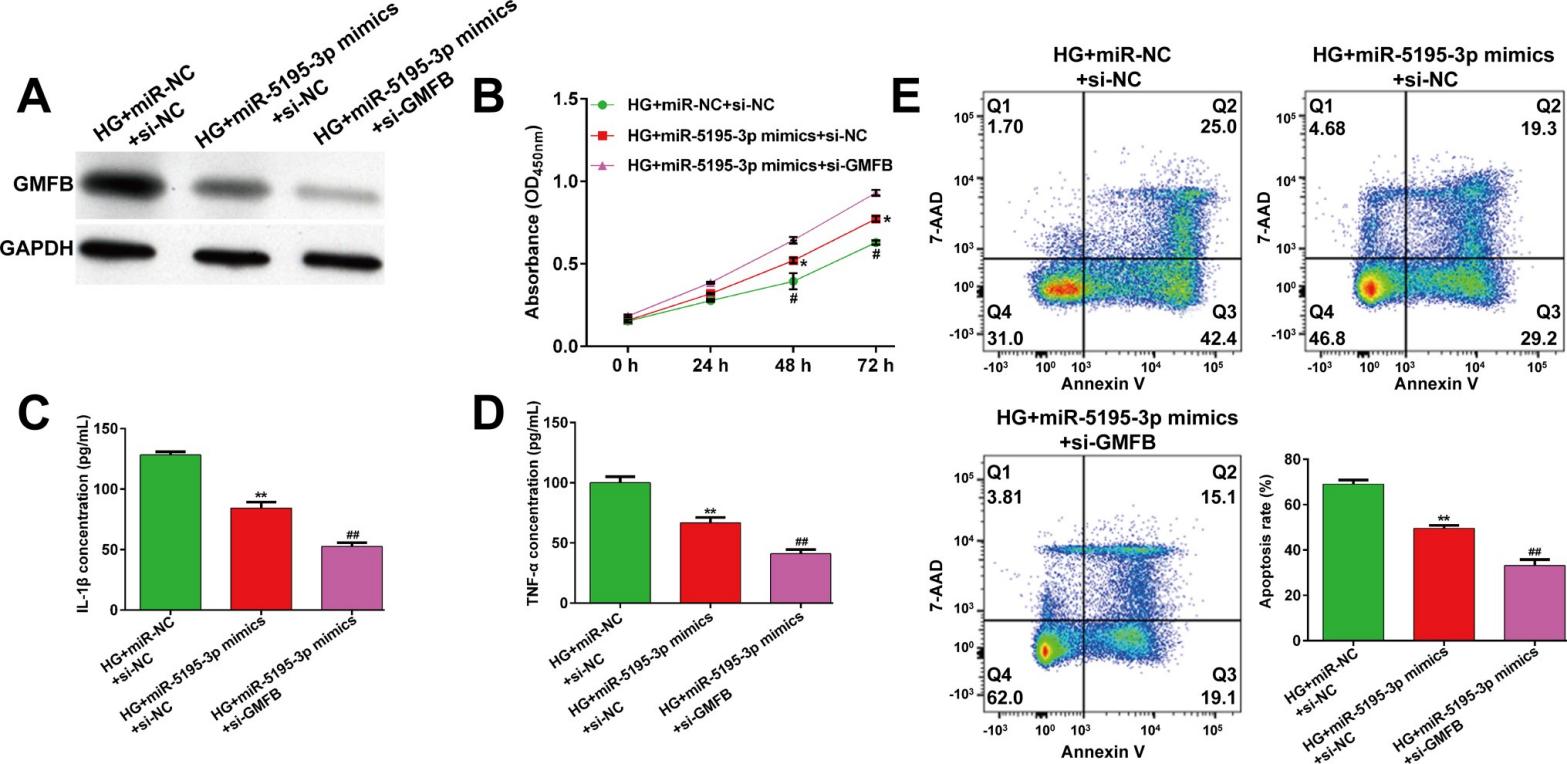
Knockdown of GMFB enhanced the protective effects of miR-5195-3p overexpression against HG-induced ARPE-19 cell injury. ARPE-19 cells were co-transfected with miR-5195-3p mimics and si-GMFB or si-NC, and then treated with HG for 24 h. (A) The protein expression of GMFB was detected by western blot analysis. (B) Cell viability was analyzed using CCK-8 assay. (C-D) ELISA assay was performed to analyze the release of IL-1β and TNF-α in transfected ARPE-19 cells, followed by HG stimulation. (E) The percentages of apoptotic cells were compared in transfected ARPE-19 cells, followed by HG stimulation. Data were shown as mean ± SD. *p < 0.05, **p < 0.01, compared with HG + miR-NC + si-NC; #*p* < 0.05, ##*p* < 0.01, compared with HG + miR-5195-3p mimics + si-NC; NC, negative control; HG, high glucose; si, small interfering.

Furthermore, we performed another rescue experiment in ARPE-19 cells by co-transfection with miR-5195-3p mimics and si-GMFB or si-NC, followed by 24h HG stimulation. Western blot analysis first showed that the downregulation of GMFB by miR-5195-3p overexpression was further decreased after co-transfection with miR-5195-3p mimics and si-GMFB in HG-stimulated ARPE-19 cells (**Fig 5A**). Subsequently, a series of functional experiments, including CCK-8 assay (**Fig 5B**), ELISA assay (**Fig 5C and 5D**) and flow cytometry analysis (**Fig 5E**) demonstrated that miR-5195-3p overexpression induced an increase in cell viability, as well as a decreased in the concentration of IL-1β and TNF-α and cell apoptosis were all enhanced after GMFB knockdown in HG-stimulated ARPE-19 cells.

## Discussion

As chronic eye complication of diabetes, DR is considered to highly associated with long-term hyperglycemia [30]. RPE cells are distributed between the choroid and the retinal neuroepithelial layer, which exert important functions in regulating intraocular ion balance, improving visual cycle metabolism and maintaining the secretion of growth-promoting factors [31]. Considering the sensibility of RPE cells to hyperglycemia [5–7], we mimicked hyperglycemia conditioning of ARPE-19 cells by exposing them to HG. Compared with NG treatment, HG induced significantly apoptosis and inflammation, as well as impaired cell viability in ARPE-19 cells. In fact, there is evidence that HG-induced aseptic inflammation is associated with the development and progression of human endothelial cell injury [32,33]. Under HG stimulation, we further discovered that miR-5195-3p expression was significantly reduced, while GMFB expression was remarkedly elevated in ARPE-19 cells. The dysregulated expression of miR-5195-3p and GMFB indicated that they might play an important role in the development of DR.

Next, we confirmed the functional role of miR-5195-3p in HG-induced cell injury by performing gain-of-function assays. Our data showed that miR-5195-3p overexpression significantly reversed impaired cell viability, increased pro-inflammatory cytokines (IL-1β and TNF-α) and cell apoptosis induced by HG stimulation. Although the studies of miR-5195-3p on DR or diabetes have not been reported, the regulatory role of miR-5195-3p on cell growth, proliferation and apoptosis has been gradually elucidated in several tumor cells. For example, miR-5195-3p sharply reduced KLF5 to suppress the proliferation and invasion of human bladder cancer cells [18]. The tumor suppressive role of miR-5195-3p was also demonstrated in non-small cell lung cancer [19], osteosarcoma [20], glioma [21], hepatocellular carcinoma [22] and colorectal cancer [34]. Moreover, miR-5195-3p could enhance the sensitivity of paclitaxel-resistant triple-negative breast cancer cells to paclitaxel treatment [35]. These reports on the anti-tumor of miR-5195-3p seem to be in line with our observations that miR-5195-3p exerted protective effects against HG-induced ARPE-19 cell injury.

Based on bioinformatics analysis prediction and luciferase reporter assay, we confirmed that GMFB was a direct target of miR-5195-3p. As expected, GMFB knockdown imitated the protective role of miR-5195-3p against HG-induced ARPE-19 cell injury. The rescue experiments further demonstrated that GMFB overexpression reversed, while knockdown enhanced the protective effects of miR-5195-3p overexpression against HG-induced ARPE-19 cell injury. These data supported that miR-5195-3p exerted protective effects against HG-induced ARPE-19 cell injury by targeting GMFB. As our best knowledge, GMFB participates in regulation of apoptosis, oxidative stress and neuroinflammation [24,25]. In recent years, GMFB has been found to be aberrantly upregulated in Alzheimer’s disease [27] and Parkinson’s disease [28]. Consistent with our data, overexpression of GMFB increased levels of reactive oxygen species and a deterioration in the state of the cells in the lung injury induced by acute cerebral ischemia [29]. We thus speculated that GMFB might be an inducer in HG-induced ARPE-19 cell injury. At present, it is worth of note that anti-VEGF agents, used in clinical practice, such as ranibizumab, bevacizumab and aflibercept are considerably different in terms of molecular interactions when they bind with VEGF [36]. These anti-VEGF agents exert anti-angiogenic activities used in retinal diseases [37]. Through our data, we may make a hypothesis that discovery of miR-5195-3p/GMFB targets can be useful to develop novel drugs exerting anti-apoptosis and anti-inflammation potentially useful in clinical practice.

## Conclusions

In summary, our study provided evidence that miR-5195-3p might alleviate HG-induced ARPE-19 cell injury by down-regulating its target gene GMFB. Our findings suggested that miR-5195-3p may serve as a promising target in regulating HG-induced ARPE-19 cell injury in DR, which provide new insights into mir-5195-3p/GMFB axis as therapeutic target for DR therapy.

## Supporting information

S1 Raw images (TIF)

## Abbreviations

DR: diabetic retinopathy
RPE: retinal pigment epithelial
GMFB: glia maturation factor-β
DMEM: Dulbecco’s Modified Eagle’s Medium
FBS: fetal bovine serum
CCK-8: Cell Counting Kit-8
3′-UTRs: 3′: untranslated regions
WT: wild type
MUT: mutant
