# Supplementary figures and images for: MicroRNA-5195-3p alleviates high glucose‑induced injury in human ARPE-19 cells by targeting GMFB
Source: PLoS One. 2021 Nov 18;16(11):e0260071. doi: 10.1371/journal.pone.0260071 (PMC8601420; doi:10.1371/journal.pone.0260071)

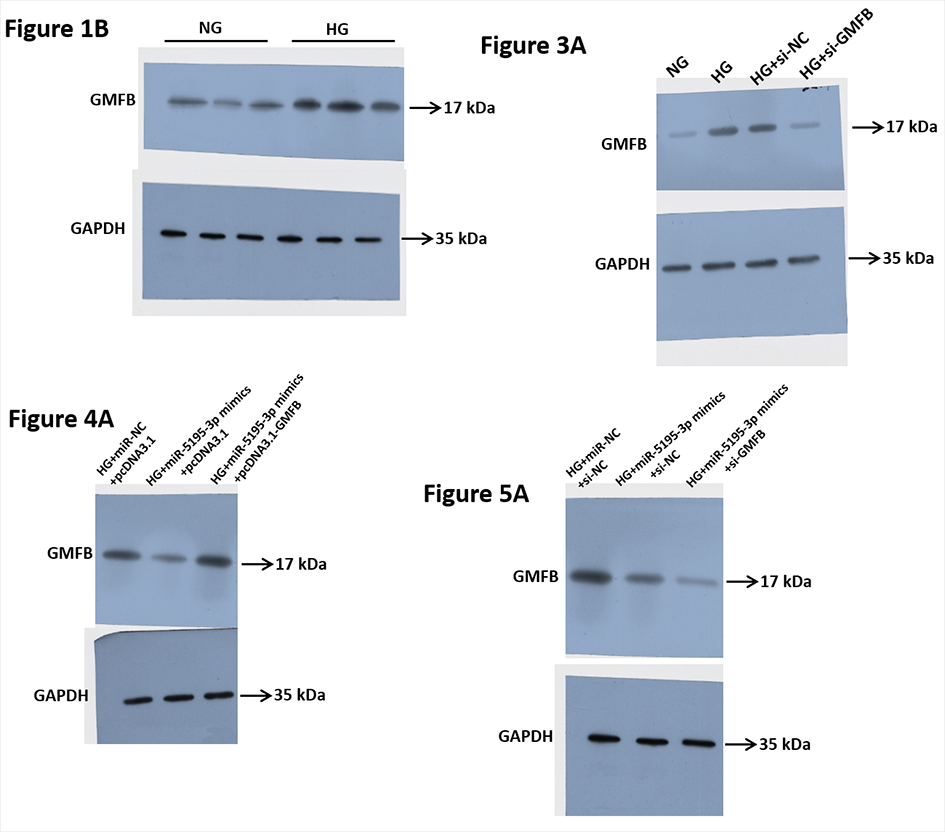

Supplement: S1 Raw images — (TIF) [file pone.0260071.s001.tif]
